# Dosimetric benefit of online treatment plan adaptation in stereotactic ultrahypofractionated MR-guided radiotherapy for localized prostate cancer

**DOI:** 10.3389/fonc.2024.1308406

**Published:** 2024-02-15

**Authors:** Christoph A. Fink, Carolin Buchele, Lukas Baumann, Jakob Liermann, Philipp Hoegen, Jonas Ristau, Sebastian Regnery, Elisabetta Sandrini, Laila König, Carolin Rippke, David Bonekamp, Heinz-Peter Schlemmer, Juergen Debus, Stefan A. Koerber, Sebastian Klüter, Juliane Hörner-Rieber

**Affiliations:** ^1^ Department of Radiation Oncology, Heidelberg University Hospital, Heidelberg, Germany; ^2^ Heidelberg Institute of Radiation Oncology (HIRO), Heidelberg, Germany; ^3^ National Center for Tumor Diseases (NCT), Heidelberg, Germany; ^4^ Institute of Medical Biometry (IMBI), University of Heidelberg, Heidelberg, Germany; ^5^ Clinical Cooperation Unit Radiation Oncology, German Cancer Research Center (DKFZ), Heidelberg, Germany; ^6^ Division of Radiology, German Cancer Research Center (DKFZ), Heidelberg, Germany; ^7^ German Cancer Consortium (DKTK), Partner Side Heidelberg, Heidelberg, Germany; ^8^ Heidelberg Ion-Beam Therapy Center (HIT), Department of Radiation Oncology, Heidelberg University Hospital, Heidelberg, Germany; ^9^ Department of Radiation Oncology, Barmherzige Brueder Hospital Regensburg, Regensburg, Germany

**Keywords:** stereotactic body radiation therapy, MR-guided radiotherapy, daily adaptive radiotherapy, prostate cancer, dosimetric benefits

## Abstract

**Background:**

Apart from superior soft tissue contrast, MR-guided stereotactic body radiation therapy (SBRT) offers the chance for daily online plan adaptation. This study reports on the comparison of dose parameters before and after online plan adaptation in MR-guided SBRT of localized prostate cancer.

**Materials and methods:**

32 consecutive patients treated with ultrahypofractionated SBRT for localized prostate cancer within the prospective SMILE trial underwent a planning process for MR-guided radiotherapy with 37.5 Gy applied in 5 fractions. A base plan, derived from MRI simulation at an MRIdian Linac, was registered to daily MRI scans (predicted plan). Following target and OAR recontouring, the plan was reoptimized based on the daily anatomy (adapted plan). CTV and PTV coverage and doses at OAR were compared between predicted and adapted plans using linear mixed regression models.

**Results:**

In 152 out of 160 fractions (95%), an adapted radiation plan was delivered. Mean CTV and PTV coverage increased by 1.4% and 4.5% after adaptation. 18% vs. 95% of the plans had a PTV coverage ≥95% before and after online adaptation, respectively. 78% vs. 100% of the plans had a CTV coverage ≥98% before and after online adaptation, respectively. The D_0.2cc_ for both bladder and rectum were <38.5 Gy in 93% vs. 100% before and after online adaptation. The constraint at the urethra with a dose of <37.5 Gy was achieved in 59% vs. 93% before and after online adaptation.

**Conclusion:**

Online adaptive plan adaptation improves target volume coverage and reduces doses to OAR in MR-guided SBRT of localized prostate cancer. Online plan adaptation could potentially further reduce acute and long-term side effects and improve local failure rates in MR-guided SBRT of localized prostate cancer.

## Highlights

- MR-guided online plan adaptation improves CTV and PTV Coverage.
- MR-guided online plan adaptation decreases dose at organs at risk.
- MR-guided online plan adaptation improves dose constraint adherence for organs at risk.

## Introduction

Radiotherapy for localized prostate cancer has undergone a remarkable evolution over the years, technical advances going along with an increasing emphasis on ultrahypofractionation (1–4). The adoption of high single dose regimens highlights the critical need for precise and accurate dose delivery to the target volume, while simultaneously adhering to strict organ at risk (OAR) constraints. Technical advances in dose optimization and delivery techniques are paving the way toward achieving this balance: The incorporation of MR-guided radiotherapy workflows has introduced a new chapter of precision and adaptability. Due to its superior soft tissue contrast, precise visualization and delineation of the prostate and neighboring anatomy is facilitated (5–7), potentially widening the therapeutic window in pelvic radiotherapy by minimizing the inadvertent irradiation of healthy tissues.

Another advantage of MR-guided radiotherapy is its capability to adapt the treatment plan according to daily anatomical variations observed at and during each session (8). Reoptimization of the daily treatment plan to deliver a base-plan-like dose distribution in each fraction may further improve efficacy and reduce toxicity (8–11). The focus of this study is to evaluate the impact of online plan adaptation on target volume coverage and OAR dose and constraint adherence in ultrahypofractionated MR-guided radiotherapy of localized prostate cancer.

## Materials and methods

### Treatment planning

We report dosimetry data of the first 32 patients at Heidelberg University Hospital from the prospective SMILE phase II trial (12) (NCT04845503). *SMILE* aims at evaluating the safety and feasibility of ultrahypofractionated radiotherapy with MR-guided radiation therapy in localized prostate cancer. All patients received MR-guided SBRT from 03/2021 to 03/2023 at Heidelberg University Hospital. The detailed treatment planning process was reported previously (12). In short, all patients underwent multiparametric MRI (mpMRI). Subsequently, a True Fast Imaging with Steady State Procession (TRUFI) sequence was utilized for a 0.35T MRI simulation scan at an MRIdian Linear Accelerator for all patients. After the MRI simulation, patients underwent a planning CT without contrast with an identical setup. A base plan was calculated based on the mpMRI and the planning CT.

### Dose specifications

Online adaptive MR-guided SBRT was administered as step-and-shoot IMRT using an MRIdian Linear Accelerator system developed by ViewRay, Inc. In cases of low-risk cancers, the clinical target volume comprised solely the prostate, while intermediate-risk cancers included the base of the seminal vesicles in the clinical target volume. The clinical target volume (CTV) was expanded uniformly by 3 mm in all directions to form the planning target volume (PTV). At least 95% of the PTV was required to receive ≥95% of the prescribed dose, with an upper limit of 107% for the maximum dose. The prescribed dose of 37.5 Gy was delivered over 5 fractions administered on alternate days. 5 patients received a simultaneous integrated boost of 40 Gy to the dominant intraprostatic lesion. A planning organ at risk volume (PRV) was created around the urethra by adding a 2 mm margin, with a dose constraint of D_0.2cc _≤37.5 Gy. For both bladder and rectum, a D_0.2cc_ ≤38.5 Gy was prescribed according to the protocol. No fiducial markers, rectal spacer gels, or other rectal devices were employed.

### Treatment

Patients were instructed to have an empty bowel before receiving radiation therapy. Additionally, patients were asked to consume 500 ml of water 30 minutes prior to the start of therapy to ensure an adequate bladder volume. Following the patient’s treatment setup, an MRI scan was conducted at the MRIdian Linear Accelerator and examined to confirm an empty rectum and satisfactory bladder filling. In a first step of adjustment, the daily MRI was matched with the base plan by translational repositioning. Subsequently, this MRI scan was registered to the MRI of the base plan based on the CTV contours. OAR contours from the planning MRI scan were deformably transferred to the daily MRI, while CTV and PTV contours were transferred rigidly. In all cases, CTV contours were adjusted by the treating physician. OARs were modified within an area around the PTV expanded by 1 cm in the cranio-caudal direction and 3 cm in all other directions on the daily MRI (13). After recontouring, the base plan was applied onto the anatomy of the day (= predicted plan). In case of any violations of either OAR dose constraints or the PTV coverage, the plan was reoptimized based on the current anatomy (= adapted plan). After reoptimization, a second MRI scan was performed to account for anatomical changes during reoptimization. In case of satisfactory anatomic alignment, the adapted plan was approved by the treating physician. On-table quality assurance was conducted using a vendor-supplied secondary dose calculation as well as an in-house software developed for evaluating target volume extension, contour accuracy and fluence modulation (14).

### Statistical analysis

The predicted and adapted treatment plans of 32 consecutive patients were analyzed to assess the coverage of CTV and PTV, as well as the radiation doses received by the rectum, urethra, and bladder as OAR. Predicted and adapted plans were analyzed based on dose volume histogram analysis considering dose constraints for OAR and target volumes as well as absolute percentages for CTV and PTV coverage. Linear mixed regression models were used to analyze dose and target coverage as continuous variables. The plan (predicted vs. adapted) was included as a fixed factor. Random intercepts for patients and days (nested within patients) were specified to account for the repeated measures. However, in many cases the estimated variance of the day-specific random intercepts was 0 and thus the random effect was removed from the respective model. 95% profile-likelihood confidence intervals (CIs) were computed for the plan differences.

When model residuals were non-normal or outliers were present, the robust variance-covariance matrix CR2 was used (15, 16). The analysis was done in R 4.3.0 using the packages lmerTest (17) and clubSandwich (18).

## Results

A total of 160 treatment sessions, consisting of five fractions per patient, were administered. Among these, plan adaptations were carried out for 152 fractions (95%). The median duration for the recontouring and the plan reoptimization was 39 minutes (range 22 – 78 minutes).

### Target volumes

Although PTV size varied from first to last adapted fraction by a median of -1.7% (range -11.2 – 11.1%), online plan reoptimization did not cause a substantial change in PTV size with a median change of 0.4% (range 0.2 – 1.3%).

**Figure 1:**
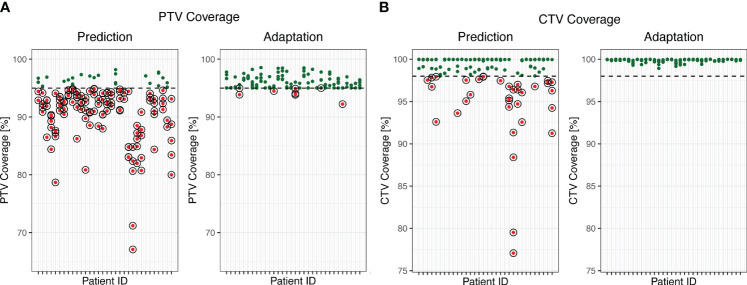
PTV **(A)** and CTV **(B)** coverage before and after online adaptation. Dots represent single fractions. Green dots meet the prespecified treatment goals. Red circled dots do not meet the prespecified treatment goals. The dashed line marks the 95% threshold **(A)** and the 98% threshold **(B)**.

Online plan adaptation yielded a 4.5% increase in mean PTV coverage (95% CI: 3.0; 6.0, p < 0.001). Regarding treatment goals, online plan adaptation enabled a PTV coverage ≥95% in 95% of the plans compared to 18% before adaptation (predicted plans), respectively (see **Figure 1A**). Regarding outliers, the patient who most profited from online plan adaptation regarding PTV coverage had a mean (median) increase of the PTV coverage of 18.9% (14.4%), whereas two patients had a slight decrease in mean (median) PTV coverage up to 1.8% (1.8%) due to OAR constraints.

The mean CTV coverage after online adaptation was 1.4% higher (95% CI: 0.5; 2.3, p = 0.004) in the adapted plans than in the predicted plans. 78% of the predicted plans had a CTV coverage ≥98%, while adaptation allowed for a CTV coverage ≥98% in all plans (see **Figure 1B**).

The mean D_95%_ increased by 0.96 Gy (95% CI: 0.49; 1.43, p < 0.001) from predicted to adapted plans. Adaptation yielded a D_95%_ of ≥35.625 Gy (95% of 37.5 Gy), in 95% of the plans compared to 18% in a non-adapted scenario, respectively. The mean D_50%_ was higher by 0.08 Gy (95% CI: 0.031; 0.133, p = 0.002) in the adapted plans than in the predicted plans. Adaptation further increased the D_50%_ of ≥37.5 Gy to 65% compared to 55% without adaptation, respectively.

### Organs at risk

**Figure 2:**
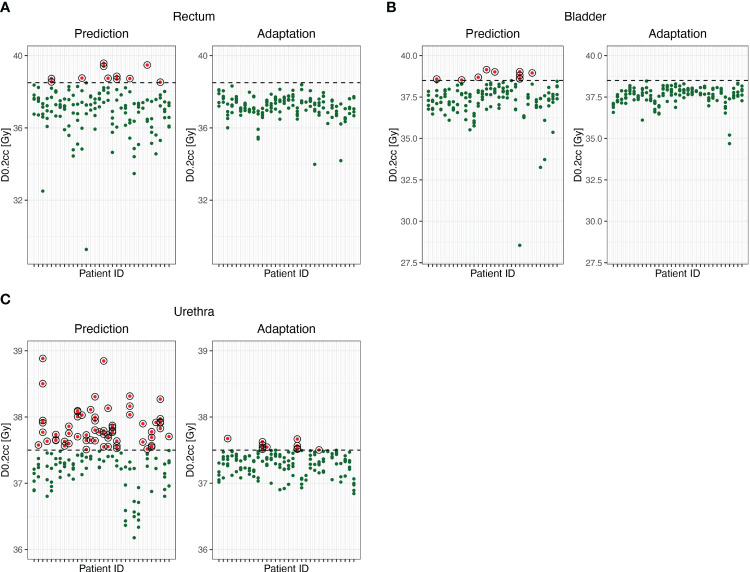
D_0.2cc_ of rectum **(A)**, bladder **(B)** and urethral PRV **(C)** before and after online adaptation. Dots represent single fractions. Green dots meet the prespecified treatment goals. Red circled dots do not meet the prespecified treatment goals.

Adaptation enabled adherence to the D_0.2cc_ for both bladder and rectum in all cases compared to 93% without adaptation (**Figures 2A, B**). Furthermore, adaptation allowed for the PRV of the urethra to meet the constraint of <37.5 Gy in 93% of the fractions compared to only 59% without adaptation (**Figure 2C**).

## Discussion

This study aimed at quantifying the effect of MR-guided online plan adaptation on relevant dose specifics in patients treated with SBRT of localized prostate cancer. In this study, online adaptation was performed by the treating physician and medical physicist in 95% of fractions. Mean PTV and CTV coverage were higher and inadvertent OAR doses lower after online plan adaptation.

In alignment with recently reported changes in prostate volume over the course of SBRT (19), PTV size varied between the first and last adapted fractions with some patients experiencing a prostate swelling while others demonstrated prostate shrinkage. However, there was no substantial change in PTV size observed through online plan adaptation.

When discussing the clinical significance of the observed effect of online adaptation, it is crucial not only to focus on the mean value deviation but also on individual patient’s single-fraction data. Kishan et al. demonstrated in their analysis of individual patient data involving over 2000 patients treated with low- and intermediate-risk prostate cancer that with a biochemical recurrence-free survival rate exceeding 90%, the cumulative long-term grade 3+ GU and GI toxicity following SBRT for localized prostate cancer is <3% and that acute toxicity acts as a risk factor for the development of late toxicity (20). The MIRAGE trial which randomized patients with localized prostate cancer to either CT- or MR-guided SBRT did not report the use of online plan adaptation techniques. Nevertheless, MIRAGE revealed that a reduction of the PTV margin in the MR-guided SBRT arm can reduce grade 2+ toxicity, with pending data on its oncological equivalence (21). However, it should be noted that some degree of grade 2+ toxicity is expected regardless of PTV margins due to the inclusion of the urethra in the CTV. In short, the majority of patients do not experience grade 2+ toxicity or local failure. Nonetheless, for those at risk, an optimized radiation plan may mitigate the risk of toxicity or local recurrence.

In line with previous dosimetric comparisons of MR-guided SBRT in localized prostate cancer (22), in this study, almost 80% of radiation plans had a CTV coverage over 98%, with nearly all plans adhering to OAR constraints before online plan adaptation, consequently resulting in a relatively low risk of toxicity. For the small fraction of cases not meeting target volume goals or OAR constraints, online adaptation could potentially affect toxicity, quality of life and failure free survival. In a recent analysis with 26 patients with localized prostate cancer, Dassen et al. compared radiation plans with separate adaptation strategies for position, rotation and shape in MR-guided online adaptive SBRT, concluding that a PTV margin reduction is probably safe in patients with inclusion of (parts of) the seminal vesicles, but not in cases of prostate-only irradiation (23). The presented dataset, illustrating the 10^th^ to 90^th^ percentile range of target volume coverage and OAR overlap, suggests variations among the plans in a similar range as presented in our study: Some radiation plans easily achieve target volume goals and OAR constraints, while others (at the lower end of the trajectories and below) may especially benefit from adaptation. Alongside individual patient risk factors such as prior cystoscopy, TUR-P, or a history of inflammatory bowel disease, there may also be planning-related risk factors, where online adaptation with plan reoptimization could play a role in minimizing the rate of low-grade toxicity and potentially eliminating high-grade toxicity (24).

Our data show that online plan adaptation may help replicating a dose distribution similar to the base plan. It further improves largely acceptable dose distributions which is of particular interest where further precision is key, i.e. with the adoption of a boost to the dominant intraprostatic lesion (2), when sparing the urethra without compromising oncological efficacy or in re-irradiation scenarios (25). Currently, it remains unclear which patients might derive the greatest advantage from online plan adaptation. For an adequate and efficient utilization of MR-guided radiotherapy in localized prostate cancer, research on both patient selection criteria and strategies to enhance workflow efficiency are warranted. Nachbar et al. have successfully trained and validated a fast, accurate deep learning model for automated MRI segmentation (26). Additionally, efforts are underway to incorporate entirely autonomous workflows, encompassing automatic OAR contouring, target delineation, and automatic planning, within the clinical setting (27). This progress could alleviate the challenges of patient selection by streamlining the workflow in MR-guided radiation therapy, especially when treatment times become more comparable to non-adaptive radiotherapy.

Limitations of our study include the small sample size and the missing estimation of the accuracy of image fusion or geometric congruence of the target volumes or OAR among the predicted and adapted plans. Dosimetric benefits may also result from enhanced alignment, which is supported by the absence of PTV size changes in median in this study. Nevertheless, PTV sizes do not truly reflect the deformation of the prostate and neither the displacement and deformation of surrounding OARs (e.g. rectum), which were often the reason for adaptation. Of note, the dosimetric benefits presented in this work are caused by daily plan adaptation after matching the current MRI with the planning MRI and hence do not result from superior alignment. This is in contrast to the MIRAGE trial, where no online plan adaptation was performed and superior toxicity results may primarily attributed to superior alignment and the possibility of gated dose delivery when compared to conventional IGRT techniques (21). A further limitation is the lack of post-adaptation MRI scan analysis for assessing potential changes in adapted plan quality due to organ motion during the replanning process. An MRI scan prior to dose delivery was performed to account for anatomical changes during reoptimization and irradiation was only started in case of satisfactory alignment. To further mitigate anatomical changes, intrafractional gating was applied. The impact of anatomical changes is currently being investigated in a separate study.

Although in CT-guided radiotherapy hyaluronic acid spacer gels have been shown to improve rectal dosimetry and hence gastrointestinal toxicity (28), this effect has not been demonstrated, to our knowledge, in MR-guided radiotherapy. Due to the occurrence of rectal fistulas after rectal spacer placement in previous trials (29), their utilization at our center has been omitted in favor of a non-interventional workflow. Nevertheless, dosimetric benefits of MR-guided online-adaptation to the rectum may be reduced by rectal spacers.

Real-time tracking of the prostate via surrogate fiducial markers may permit a similar reduction in PTV margins (30). However, fiducial marker implantation is an invasive procedure accompanied by a slightly increased risk for infection or even fiducial migration in rare cases which might lead to impaired tracking (31). Furthermore, the application of radiopaque fiducials enables rigid-registration and therefore does provide limited information on organ deformation, seminal vesicle location, or bladder and rectal distension. A deviation in the shape of the prostate may not be effectively corrected by standard IGRT applications with or without the use of fiducials. This again underlines the need for deformable image registration and adaptive planning in prostate SBRT at least for some patients (32).

In conclusion, this study demonstrates that online plan adaptation improves target volume coverage and reduces doses at OAR in MR-guided SBRT of localized prostate cancer. This could potentially further reduce acute and long-term side effects and improve local failure rates. Future research may focus on identifying subgroups of patients that particularly gain significant clinical benefit from the online adaptation process as well as automatic planning efforts to streamline MR-guided radiotherapy workflows.

## Data availability statement

The raw data supporting the conclusions of this article will be made available by the authors, without undue reservation.

## Ethics statement

The studies involving humans were approved by ethics committee of the University Hospital Heidelberg. The studies were conducted in accordance with the local legislation and institutional requirements. The participants provided their written informed consent to participate in this study.

## Author contributions

CF: Conceptualization, Data curation, Formal analysis, Investigation, Methodology, Visualization, Writing – original draft, Writing – review & editing. CB: Conceptualization, Data curation, Formal analysis, Investigation, Methodology, Software, Visualization, Writing – original draft, Writing – review & editing. LB: Data curation, Formal analysis, Methodology, Software, Validation, Writing – original draft, Writing – review & editing. JL: Data curation, Investigation, Writing – review & editing. PH: Writing – review & editing, Data curation. JR: Data curation, Investigation, Writing – review & editing. SR: Data curation, Investigation, Writing – review & editing. ES: Data curation, Writing – review & editing. LK: Data curation, Writing – review & editing. CR: Data curation, Writing – review & editing. DB: Data curation, Investigation, Methodology, Resources, Writing – review & editing. H-PS: Data curation, Investigation, Methodology, Resources, Writing – review & editing. JD: Conceptualization, Formal analysis, Investigation, Supervision, Writing – review & editing. SAK: Conceptualization, Data curation, Investigation, Supervision, Writing – review & editing. SK: Conceptualization, Data curation, Investigation, Supervision, Writing – review & editing. JH-R: Conceptualization, Data curation, Investigation, Methodology, Supervision, Visualization, Writing – original draft, Writing – review & editing.
